# The Effect of Anodal Transcranial Direct Current Stimulation Over Left and Right Temporal Cortex on the Cardiovascular Response: A Comparative Study

**DOI:** 10.3389/fphys.2018.01822

**Published:** 2018-12-18

**Authors:** Luca Angius, Samuele M. Marcora, James G. Hopker, Alexis R. Mauger

**Affiliations:** Endurance Research Group, School of Sport and Exercise Sciences, University of Kent, Kent, United Kingdom

**Keywords:** tDCS, heart rate, cardiovascular, metaboreflex, cardiac output, autonomic nervous system

## Abstract

**Background:** Stimulation of the right and left anterior insular cortex, increases and decreases the cardiovascular response respectively, thus indicating the brain’s lateralization of the neural control of circulation. Previous experiments have demonstrated that transcranial direct current stimulation (tDCS) modulates the autonomic cardiovascular control when applied over the temporal cortex. Given the importance of neural control for a normal hemodynamic response, and the potential for the use of tDCS in the treatment of cardiovascular diseases, this study investigated whether tDCS was capable of modulating autonomic regulation.

**Methods:** Cardiovascular response was monitored during a post-exercise muscle ischemia (PEMI) test, which is well-documented to increase sympathetic drive. A group of 12 healthy participants performed a PEMI test in a control (Control), sham (Sham) and two different experimental sessions where the anodal electrode was applied over the left temporal cortex and right temporal cortex with the cathodal electrode placed over the contralateral supraorbital area. Stimulation lasted 20 min at 2 mA. The hemodynamic profile was measured during a PEMI test. The cardiovascular parameters were continuously measured with a transthoracic bio-impedance device both during the PEMI test and during tDCS.

**Results:** None of the subjects presented any side effects during or after tDCS stimulation. A consistent cardiovascular response during PEMI test was observed in all conditions. Statistical analysis did not find any significant interaction and any significant main effect of condition on cardiovascular parameters (all *p*s > 0.316) after tDCS. No statistical differences regarding the hemodynamic responses were found between conditions and time during tDCS stimulation (*p* > 0.05).

**Discussion:** This is the first study comparing the cardiovascular response after tDCS stimulation of left and right TC both during exercise and at rest. The results of the current study suggest that anodal tDCS of the left and right TC does not affect functional cardiovascular response during exercise PEMI test and during tDCS. In light of the present and previous findings, the effect of tDCS on the cardiovascular response remains inconclusive.

## Introduction

During exercise, central command (a feedforward mechanism) and the exercise pressor reflex (a feedback mechanism) send signals that converge in the cardiorespiratory centers located in the medulla (Mitchell et al., 1983; Williamson et al., 2006; Matsukawa, 2012). Both mechanisms contribute to the shift of the sympathetic drive to increase the cardiovascular response (Williamson et al., 2006; Matsukawa, 2012), resulting in an elevation of cardiac output (CO), systemic vascular resistance (SVR), and mean arterial blood pressure (MAP) (Lewis et al., 1983). Particular attention has been given to the neurocircuitry involved in the cardiovascular regulation during exercise. Cortical and subcortical areas of the brain such as the insula cortex (IC), anterior cingular cortex (ACC), thalamus, hypothalamus, amygdala, and medial prefrontal region have been well-documented as participating in the regulation of the cardiovascular system during exercise (Benarroch, 1993; Williamson et al., 2006; Cechetto and Shoemaker, 2009), with the ACC and IC primarily involved during the activation of the exercise pressor reflex (Williamson et al., 1999, 2006; Sander et al., 2010; Basnayake et al., 2012; Williams et al., 2013).

In order to identify the level of cortical control of the heart, experiments involving deep brain stimulation have been performed. When stimulated, the left IC has been shown to decrease the cardiovascular response, while stimulation of the right IC has the opposite effect, thus supporting the assumption of a cortical lateralization of the brain regarding cardiovascular control (Oppenheimer et al., 1992a,b). In agreement, similar conclusions have been proposed in experiments involving patients affected by lesions on the left or right IC, epilepsy and post-stroke damage (Sander and Klingelhöfer, 1994; Oppenheimer et al., 1996).

Recently non-invasive techniques such as the transcranial direct current stimulation (tDCS) and repeated transcranial magnetic stimulation (rTMS) have been demonstrated to induce changes in activation of the targeted brain area by increasing or decreasing their activity (Nitsche and Paulus, 2001; Nitsche et al., 2005; Fitzgerald et al., 2006; Filmer et al., 2014; Rossini et al., 2015; Polanía et al., 2018). Given the specificity of some cortical areas for the cardiovascular regulation, the application of non-invasive techniques can be used to study their effect on the cardiovascular response as a potential and useful tool to be explored. Non-invasive brain stimulation techniques have been proposed as a therapy given their ability to target specific brain networks, particularly as pharmacologic treatments have significant limitations, including difficulty to concentrate the medication on the tissues of interest. The efficacy of other treatments, such as physical exercise, largely depend on the expertise of the therapist.

Transcranial direct current stimulation has previously been used to relieve pain (Boggio et al., 2008) and treat neurological or psychiatric disorders (Fregni et al., 2007). Moreover, its effects are not only limited to the targeted areas under the scalp but also to subcortical areas. In fact, studies involving anodal stimulation (which increases the activity of the targeted area) over the temporal cortex (TC) showed alteration of heart rate variability (HRV) (Montenegro et al., 2011) and reduction of heart rate (HR) during cycling exercise (Okano et al., 2015). Despite the promising evidence regarding the ability to manipulate a targeted brain area, the number of studies investigating the application of non-invasive brain techniques on the cardiovascular response is surprisingly very limited, with no studies comparing the effect of anodal tDCS on the right and left TC. A review from Cogiamanian et al. (2010) proposed a novel therapy in the management of cardiovascular diseases by applying non-invasive brain stimulation techniques to patients. The regulation of cardiovascular control at rest or during exercise in both in healthy and clinical populations is important and thus non-invasive brain techniques might in part be used to manage cardiovascular problems such as hypertension. Despite the increasing evidence of the effects of non-invasive brain stimulation on the cardiovascular system, the majority of existing studies have been designed to evaluate the safety of these techniques on cardiovascular parameters in clinical populations (Schestatsky et al., 2013; Makovac et al., 2017). Accordingly, given the potential benefits of tDCS in the treatment of cardiovascular diseases, we monitored multiple cardiovascular variables following tDCS over both the left and right TC in a group of healthy volunteers. The aim of the present experiment was to elucidate whether the hypothesized tDCS induced alteration of sympathetic and parasympathetic activity might subsequently alter the cardiovascular response.

## Materials and Methods

### Participants and Experimental Design

**FIGURE 1:**
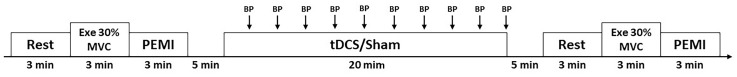
Overall view of the experimental protocol during resting condition (Rest), during intermittent handgrip exercise (Exe) and post-exercise muscle ischemia (PEMI) in all conditions. BP, blood pressure measurement; MVC, maximal voluntary contraction.

Twelve recreationally active, healthy volunteers (six males and six females), aged 21.8 ± 2.6 year, height 175 ± 11 cm and weight of 75.5 ± 17.8 kg were recruited. Previous studies involved a sample size ranging from 9 to 11 participants (Roberto et al., 2012; Crisafulli et al., 2013) which was able to detect changes in hemodynamic response during the same exercise protocol required for this experiment, and have been used in previous studies involving tDCS (Montenegro et al., 2013; Okano et al., 2015; Angius et al., 2018). All participants were engaging in at least 3 min × 60 min bouts of exercise per week at the time of the study. None of the participants reported any history of cardiovascular, pulmonary or metabolic disorders or were taking any medication during the study. All participants were asked to refrain from exercise, caffeine and alcohol intake in the 24 h prior to each visit. The study was approved by the Institutional Ethics Committee (University of Kent) according to the Declaration of Helsinki. The study followed a single-blind, randomized cross-over experimental design, and participants visited the laboratory on five separate occasions at the same time of day, separated by at least 72 h. The protocol involved two post-exercise muscle ischemia (PEMI) sessions interspaced by 20 min of tDCS stimulation (Figure 1). All experiments were carried out in a temperature-controlled (20°C, humidity 50%), air-conditioned, quiet room.

### Post-exercise Muscle Ischemia (PEMI)

This test involved a 3 min rest period, followed by 3 min of exercise consisting of dynamic rhythmic handgrip contractions at 30% of the participants’ maximal voluntary contraction (MVC). The MVC was assessed at the start of each experimental visit and was recorded as the peak force achieved over three maximal handgrip contractions on hydraulic dynamometer (MAP 1.1; Kern & Sohn, Balingen, Germany). Rhythmic contractions were guided by an electronic metronome at a rate of 30 compressions/min (contraction relaxation ratio 1:1). During the execution of the test, visual feedback of the force produced on the dynamometer was given. In addition, a metronome was used to pace the contraction. To obtain an indirect measure of central command (Williamson et al., 2003, 2006), during the rhythmic contractions participants reported their rating of perceived exertion (RPE) at the end of each minute of exercise using the Borg scale (Borg, 1998). After 3 min of exercise, a cuff was rapidly inflated (<3 s) to 50 mmHg above exercise systolic pressure on the exercising arm using an automated pneumatic device (Hokanson E20 Rapid Cuff Inflator and AG101 Air Source, Bellevue, WA, United States). The cuff was kept inflated for 3 min, after which it was then deflated. PEMI has been well-documented to trap the metabolites in the exercising muscles to maintain the stimulation of the metabo-receptors (Roberto et al., 2012; Crisafulli et al., 2013).

### tDCS Procedures

Transcranial direct current stimulation was delivered by a direct current stimulator (TCT Research Limited, Hong Kong) using a pair of humidified sponges (4 cm × 3 cm) in a water saline solution. Electric current was delivered at an intensity of 2 mA for 20 min. For the left condition (Left TC), the anodal electrode was applied over the left TC on the T3 area according to the international standards for EEG 10–20 system, with the cathodal electrode placed over the contralateral supraorbital area (Fp2). For the right condition (Right TC), the anodal electrode was applied over the right TC on the T4 area, with the cathodal electrode placed over the contralateral supraorbital area (Fp3). T3 and T4 areas were located at 40% of the distance from the Cz point to the pre-auricular point, according to the international standards for EEG 10–20. These procedures were strictly followed during each experimental condition in order to reduce the influence of extrinsic factors that might potentially affect the quality of the stimulation. For the Sham condition, electrodes were applied in the same position as the Left TC, but stimulation lasted only 30 s, after which it was rapidly ramped down. This allowed the researchers to mimic the initial sensations commonly experienced with active tDCS but without providing any change in cortical excitability (Boggio et al., 2008; Mylius et al., 2012). This procedure allowed the participants to remain “blind” in respect to the type of stimulation received during the Left TC and Right TC condition and to assure a Sham control effect. In all conditions, the stimulator was placed in such a way that subjects were unable to view the settings of the parameters of the stimulator. Participants were unable to identify the difference between the Left TC, Right TC, and Sham conditions. No electrodes were placed during the control condition (Control) and instead participants sat quietly for 20 min. To ensure a proper quality of the stimulation, electrode sponges were soaked with standard saline solution (NaCl 9%) and elasticated straps maintained electrode location. The resistance was constantly monitored on the stimulator’s display within a range between 4 and 5 kΩ. The electrode montage used for this experiment has been applied in previous experiments (Montenegro et al., 2011; Okano et al., 2015), demonstrating the ability to reach both cortical and subcortical brain areas and induce changes in the autonomic control to the heart in active healthy volunteers.

### Hemodynamic Assessment

Stroke volume (SV), HR, CO, SV/LVET ratio (stroke volume/left ventricular ejection time ratio) and SVR were monitored during all phases of the experiment with a transthoracic bioimpedance device (Physioflow PF05L1, Manatec, Petit-Ebersviller, France) that allows continuous, non-invasive monitoring of hemodynamic parameters. The method has been previously described by Charloux et al. (2000). Electrodes (Ambu Blue Sensor VL, Ambu A/S, Ballerup, Denmark) were placed over the chest in the V1 and V6 positions to the left ventricle to obtain an ECG signal, and then on the back in the midpoint of the spine corresponding to the same vertical position as the xiphoid process. Skin areas were shaved and cleaned in order to minimize electrical impedance. The PhysioFlow was calibrated during each experimental session before the tests. Systolic arterial pressure (SAP), diastolic arterial pressure (DAP), MAP was measured every minute during PEMI, and every 2 min during tDCS stimulation. Arterial blood pressure parameters were obtained by an automated blood pressure device (Tango+, SunTech Medical, Morrisville, NC, United States) (Cameron et al., 2004; Hartwich et al., 2011; Pageaux et al., 2015) with a set of three electrodes placed in V2, V6, and RL positions. The cuff was placed on the left arm of the subject. Hemodynamic measurement was later included for six subjects while HR, SAP, DAP, and MAP were monitored for all 12 subjects. MAP was calculated using the following equation:

$$\text{MAP}:\frac{(2\cdot \text{DAP})+\text{SAP}}{3}$$

### Data and Statistical Analysis

All data are presented as mean ± SD. Beat-to-beat hemodynamic and RPE collected data were averaged for 3 min during both PEMI tests. Beat-to-beat hemodynamic collected data during the 20 min of tDCS stimulation were averaged for the last min every 2 min.

Unless specified, data are presented as mean ± SD. Assumption of statistical tests such as normal distribution was checked by using the Shapiro–Wilk and sphericity of data was checked by using the Mauchly’s test. The Greenhouse–Geisser correction to the degrees of freedom was applied when violations to sphericity were present. Fully repeated measures three-way ANOVAs were used to monitor the effect of condition (control, Sham, Right TC, and Left TC), test (pre vs. post) and time (Rest, Exe, and PEMI) on the hemodynamic and perceptive data collected during both PEMI tests. Fully repeated two-way ANOVAs were performed to monitor the effect of condition (control, Sham, right TC, and left TC) and time (1, 3, 5, 7, 9, 11, 13, 15, 17, 19 min) on the hemodynamic data collected during the 20 min of tDCS stimulation. Statistical analyses were followed by Bonferroni *post hoc* when appropriate. The Statistical Package for the Social Sciences (IBM, SPSS Statistics 20.0) was used to perform all analysis, and all test assumptions were met. Statistical significance was set as *p* < 0.05 in all cases. All data are presented as means ± SD.

## Results

None of the subjects presented any side effects during or after tDCS stimulation. All subjects reported feeling an itching sensation during the Sham condition and none of the participants could tell the difference between Sham and the actual tDCS stimulation. Tables 1, 2 shows absolute values of hemodynamic variables collected during all the phases of the experiment. There were no statistical differences at Rest between each experimental condition for all the hemodynamic parameters (*p* > 0.05).

**Table 1:** Hemodynamic variables during Rest, Exe, and PEMI periods in both Control and Sham conditions.

	Control		Sham	
	Pre	Post	Pre	Post
***SV (ml)***				
Rest	83.5 ± 4.2	84.9 ± 5.2	82.2 ± 5.0	83.4 ± 6.5
Exe	92.2 ± 4.9^∗^	93.7 ± 5.7^∗^	94.0 ± 4.9^∗^	94.1 ± 4.9^∗^
PEMI	93.8 ± 6.3^∗^	94.9 ± 4.8^∗^	90.4 ± 4.5^∗^	92.3 ± 3.8^∗^
***CO (l⋅min^−1^)***				
Rest	6.0 ± 0.3	6.1 ± 0.6	5.8 ± 0.7	5.8 ± 0.7
Exe	7.5 ± 0.9^∗^	7.6 ± 1.0^∗^	7.5 ± 0.8^∗^	7.5 ± 0.9^∗^
PEMI	7.1 ± 0.6^∗^	7.2 ± 0.8^∗^	6.8 ± 0.8^∗^	6.9 ± 0.8^∗^
***SV/LVET***				
Rest	0.2 ± 0.0	0.2 ± 0.0	0.2 ± 0.0	0.2 ± 0.0
Exe	0.2 ± 0.0^∗^	0.2 ± 0.0^∗^	0.2 ± 0.0^∗^	0.2 ± 0.0^∗^
PEMI	0.2 ± 0.0^∗^	0.2 ± 0.0^∗^	0.25 ± 0.0^∗^	0.2 ± 0.0^∗^
***SVR (dyne⋅s^−1^⋅cm^5^)***				
Rest	1085 ± 97	1059 ± 153	1118 ± 270	1129 ± 164
Exe	945 ± 140^∗^	942 ± 146^∗^	930 ± 204^∗^	962 ± 153^∗^
PEMI	1022 ± 137	1002 ± 157	1093 ± 168	1055 ± 171
***SAP (mmHg)***				
Rest	107.8 ± 12.2	107.1 ± 11.8	106.1 ± 12.6	103.3 ± 7.6
Exe	114.2 ± 13.3^∗^	115.8 ± 14.4^∗^	113.6 ± 16.6^∗^	113.7 ± 9.1^∗^
PEMI	114.8 ± 16.1^∗^	115.8 ± 16.5^∗^	112.5 ± 16.3^∗^	111.7 ± 8.3^∗^
***DAP (mmHg)***				
Rest	62.6 ± 7.7	61.3 ± 7.2	62.9 ± 9.5	63.5 ± 8.5
Exe	66.1 ± 8.8^∗^	66.0 ± 8.0^∗^	67.8 ± 11.1^∗^	67.5 ± 9.7^∗^
PEMI	67.5 ± 9.6^∗^	66.9 ± 8.3^∗^	67.8 ± 11.0^∗^	67.7 ± 10.6^∗^

*Rest, resting condition; Exe, exercise; PEMI, post-exercise muscle ischemia; SV, stroke volume; CO, cardiac output; SVR, systemic vascular resistance; SV/LVET, stroke volume left ventricular ejection time ratio; SAP, systolic arterial pressure; DAP, diastolic arterial pressure; ^∗^p < 0.05 vs. Rest. Data are presented as means ± SD (n = 12).*

**Table 2:** Hemodynamic variables during Rest, Exe, and PEMI periods in both Control and Sham conditions.

	Right TC		Left TC	
	Pre	Post	Pre	Post
***SV (ml)***				
Rest	82.3 ± 8.0	82.5 ± 6.2	79.9 ± 4.7	80.5 ± 3.1
Exe	94.7 ± 4.4^∗^	94.8 ± 5.1^∗^	94.4 ± 3.5^∗^	95.6 ± 4.7^∗^
PEMI	93.4 ± 3.7^∗^	94.9 ± 4.2^∗^	92.6 ± 3.3^∗^	93.8 ± 4.4
***CO (l⋅min^−1^)***				
Rest	5.8 ± 0.8	5.8 ± 0.6	5.6 ± 0.2	5.6 ± 0.2
Exe	7.6 ± 0.8^∗^	7.7 ± 0.8^∗^	8.0 ± 0.5^∗^	8.1 ± 0.5^∗^
PEMI	6.9 ± 0.9^∗^	7.0 ± 0.8^∗^	7.6 ± 0.7^∗^	7.5 ± 0.7^∗^
***SV/LVET***				
Rest	0.2 ± 0.0	0.2 ± 0.0	0.2 ± 0.0	0.2 ± 0.0
Exe	0.2 ± 0.0^∗^	0.2 ± 0.0^∗^	0.2 ± 0.0^∗^	0.2 ± 0.0^∗^
PEMI	0.2 ± 0.0^∗^	0.2 ± 0.0^∗^	0.2 ± 0.0^∗^	0.2 ± 0.0^∗^
***SVR (dyne⋅s^−1^⋅cm^5^)***				
Rest	1146 ± 156	1185 ± 176	1245 ± 74	1191 ± 74
Exe	953 ± 106^∗^	965 ± 132^∗^	934 ± 20^∗^	891 ± 25^∗^
PEMI	1078 ± 166	1053 ± 161	986 ± 97	1001 ± 98
***SAP (mmHg)***				
Rest	110.1 ± 15.3	109.7 ± 15.1	111.5 ± 14.1	109.3 ± 15.3
Exe	115.8 ± 20.7^∗^	116.1 ± 21.2^∗^	117.4 ± 19.5^∗^	117.5 ± 19.5^∗^
PEMI	116.2 ± 21.6^∗^	114.9 ± 21.1^∗^	116.8 ± 20.5^∗^	115.8 ± 20.3^∗^
***DAP (mmHg)***				
Rest	60.7 ± 6.7	62.5 ± 8.1	62.5 ± 8.5	63.8 ± 7.4
Exe	66.2 ± 8.7^∗^	66.4 ± 11.8^∗^	68.0 ± 7.9^∗^	68.1 ± 7.8^∗^
PEMI	67.2 ± 9.4^∗^	66.2 ± 10.0^∗^	68.5 ± 9.1^∗^	68.5 ± 8.6^∗^

*Rest, resting condition; Exe, exercise; PEMI, post-exercise muscle ischemia; SV, stroke volume; CO, cardiac output; SVR, systemic vascular resistance; SV/LVET, stroke volume left ventricular ejection time ratio; SAP, systolic arterial pressure; DAP, diastolic arterial pressure; ^∗^p < 0.05 vs. Rest. Data are presented as means ± SD (n = 12).*

### Hemodynamic Response During PEMI

**FIGURE 2:**
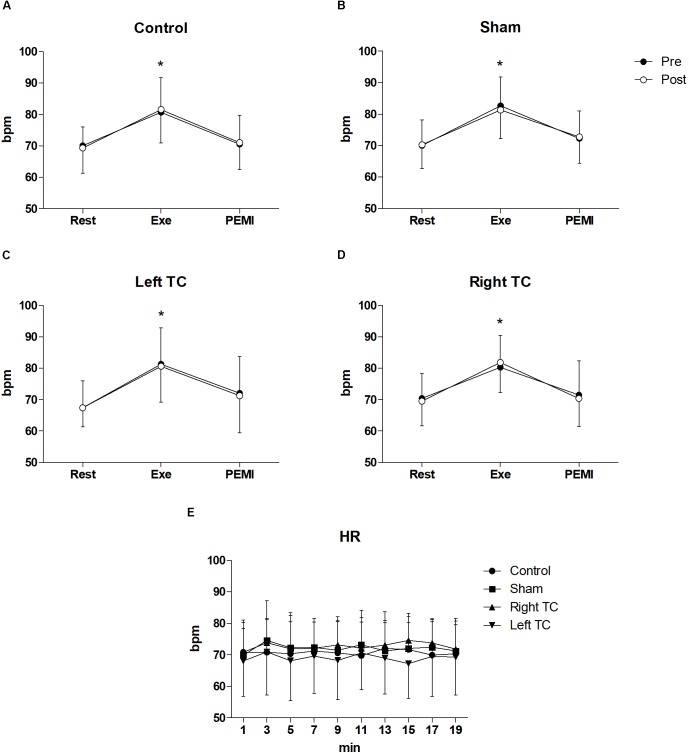
Time courses of heart rate (HR) response during the various phases of the experiment. **(A–D)** Shows time courses of HR, at rest (Rest), during exercise (Exe) and PEMI in all conditions. **(E)** Shows HR response during stimulation. Data were averaged over 3 min. ^∗^*p* < 0.05 vs. rest and PEMI. Data are presented as mean ± SD (*n* = 12).

**FIGURE 3:**
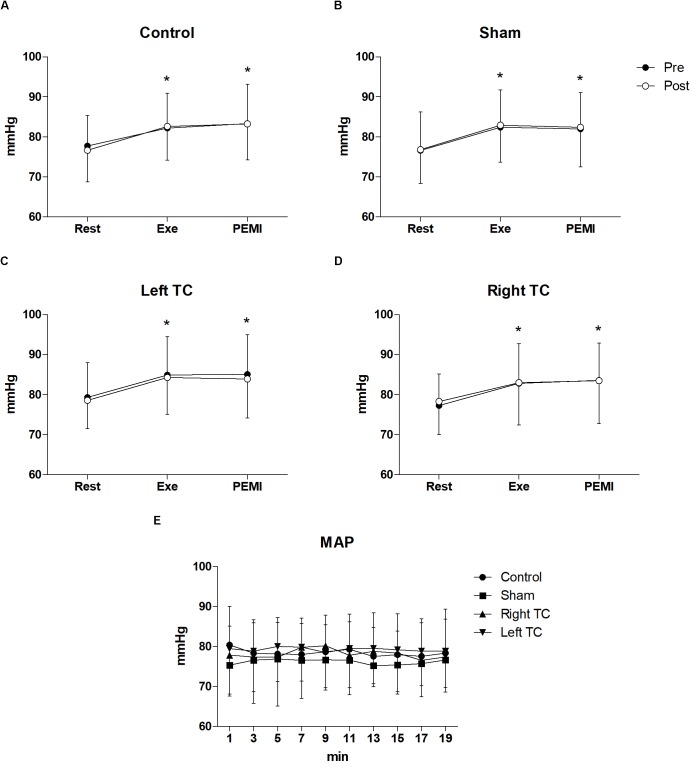
Time courses of mean arterial pressure (MAP) during the various phases of the experiment. **(A–D)** Shows time courses of MAP, at resting condition (Rest), during exercise (Exe) and PEMI in all conditions. **(E)** Shows MAP response during stimulation. ^∗^*p* < 0.05 vs. rest. Data are presented as mean ± SD (*n* = 12).

Statistical analysis did not show any differences regarding all the hemodynamic parameters between conditions during both PEMI. In details for HR *F*_(3,33)_ = 0.877, *p* = 0.463, $\mu_{p}^{2}$ = 0.74, *F*_(1,11)_ = 0.774, *p* = 0.398, $\mu_{p}^{2}$ = 0.066, for SV *F*_(3,15)_ = 2.3219, *p* = 0.128, $\mu_{p}^{2}$ = 0.307, *F*_(1,5)_ = 2.382, *p* = 0.183, $\mu_{p}^{2}$ = 0.323, for CO *F*_(3,15)_ = 2.299, *p* = 0.119, $\mu_{p}^{2}$ = 0.315, *F*_(1,5)_ = 0.001, *p* = 0.976, $\mu_{p}^{2}$ = 0.000, for SV/LVET *F*_(3,15)_ = 0.358, *p* = 0.784, $\mu_{p}^{2}$ = 0.67, *F*_(1,5)_ = 0.615, *p* = 0.468, $\mu_{p}^{2}$ = 0.110, for SVR *F*_(3,15)_ = 2.692, *p* = 0.830, $\mu_{p}^{2}$ = 0.350, *F*_(1,5)_ = 0.093, *p* = 0.773, $\mu_{p}^{2}$ = 0.018, for SAP *F*_(3,15)_ = 1.143, *p* = 0.346, $\mu_{p}^{2}$ = 0.094, *F*_(1,11)_ = 0.406, *p* = 0.537, $\mu_{p}^{2}$ = 0.036, for DAP *F*_(3,15)_ = 0.569, *p* = 0.639, $\mu_{p}^{2}$ = 0.049, *F*_(1,11)_ = 0.015, *p* = 0.904, $\mu_{p}^{2}$ = 0.001, for MAP *F*_(3,15)_ = 0.917, *p* = 0.443, $\mu_{p}^{2}$ = 0.77, *F*_(1,11)_ = 0.032, *p* = 0.861, $\mu_{p}^{2}$ = 0.003. A normal hemodynamic profile response was observed during PEMI tests in all conditions. HR was elevated during Exe in all conditions compared to Rest and then returned to Rest values during the occlusion (*F*_(3,15)_ = 2.32, *p* = 0.001, $\mu_{p}^{2}$ = 0.937, Figure 2). The following parameters increased during Exe and PEMI compared to Rest: SV (*F*_(3,15)_ = 15.58, *p* = 0.002, $\mu_{p}^{2}$ = 0.974), CO (*F*_(3,15)_ = 148.49, *p* = 0.001, $\mu_{p}^{2}$ = 0.959), SV/LVET ratio (*F*_(3,33)_ = 15.58, *p* = 0.001, $\mu_{p}^{2}$ = 0.991), SAP (*F*_(3,33)_ = 17.63, *p* = 0.001, $\mu_{p}^{2}$ = 0.744), DAP (*F*_(3,33)_ = 15.58, *p* = 0.001, $\mu_{p}^{2}$ = 0.744), MAP (*F*_(3,33)_ = 19.08, *p* = 0.001, $\mu_{p}^{2}$ = 0.778) significantly rose compared to Rest during both exercise and PEMI in all conditions (*p* < 0.05), as shown in Figure 3. SVR (*F*_(3,15)_ = 18.39, *p* = 0.001, $\mu_{p}^{2}$ = 0.980) significantly decreased during exercise and occlusion compared to Rest state.

### Hemodynamic Response During tDCS Stimulation

Statistical analysis did not show any significant interaction nor differences between conditions and time for all the hemodynamic parameters between conditions during tDCS stimulation. In details, HR (*F*_(3,33)_ = 0.898, *p* = 0.453, $\mu_{p}^{2}$ = 0.075, *F*_(3,33)_ = 0.960, *p* = 0.447, $\mu_{p}^{2}$ = 0.080), SV (*F*_(3,15)_ = 0.270, *p* = 0.846, $\mu_{p}^{2}$ = 0.051, *F*_(3,15)_ = 1.192, *p* = 0.323, $\mu_{p}^{2}$ = 0.193), CO (*F*_(3,15)_ = 0.480, *p* = 0.701, $\mu_{p}^{2}$ = 0.088, *F*_(3,15)_ = 1.083, *p* = 0.394, $\mu_{p}^{2}$ = 0.178), SV/LVET ratio (*F*_(3,15)_ = 0.652, *p* = 0.594, $\mu_{p}^{2}$ = 0.115, *F*_(3,15)_ = 0.383,*p* = 0.937, $\mu_{p}^{2}$ = 0.071), SVR (*F*_(3,15)_ = 0.644, *p* = 0.599, $\mu_{p}^{2}$ = 0.114, *F*_(3,15)_ = 0.627, *p* = 0.768, $\mu_{p}^{2}$ = 0.111), SAP (*F*_(3,33)_ = 1.874, *p* = 0.153, $\mu_{p}^{2}$ = 0.146, *F*_(3,15)_ = 0.920, *p* = 0.512, $\mu_{p}^{2}$ = 0.077), DAP (*F*_(3,33)_ = 1.191, *p* = 0.328, $\mu_{p}^{2}$ = 0.98, *F*_(3,15)_ = 0.395, *p* = 0.935, $\mu_{p}^{2}$ = 0.035), MAP (*F*_(3,33)_ = 2.024, *p* = 0.130, $\mu_{p}^{2}$ = 0.155, *F*_(3,15)_ = 0.659, *p* = 0.744, $\mu_{p}^{2}$ = 0.057) (Figures 2, 3).

## Discussion

This study sought to elucidate whether tDCS of left and right TC caused changes to the cardiovascular response during rest, exercise, and PEMI. We hypothesized that anodal tDCS of both left and right TC would alter the cardiovascular response both at rest and during exercise. However, the primary finding of the present study was that tDCS did not alter any of the cardiovascular parameters measured. The hemodynamic profile observed during exercise and PEMI is in good agreement with previous findings (Crisafulli et al., 2006, 2013; Roberto et al., 2012). As expected, during exercise and PEMI, cardiac activity significantly increased compared to baseline showing a substantial increase in SV and CO with SAP, DAP and MAP, while SVR and SV/LVET decreased. These data further support the concept that metaboreflex activation achieved by PEMI is able to stimulate both a central and peripheral cardiovascular response despite the absence of central command (Crisafulli et al., 2006, 2013; Boushel, 2010; Roberto et al., 2012). It should be noted that HR was not affected during the PEMI maneuvre, and instead returned toward baseline. The likely reason for this response is due to the pronounced vagal tone, despite the persistent sympathetic activity (Stramba-Badiale et al., 1991; Tulppo et al., 1998).

Previous research has suggested a modulation of cardiovascular response following stimulation of a specific brain area using non-invasive techniques such as rTMS and tDCS. Yoshida et al. (2001) found a transient increase in HRV following low frequency rTMS over the vertex, while Hong et al. (2002) showed a temporary reduction of blood pressure in rats following unilateral stimulation of the motor cortex, thus supporting a potential activation of the para-sympathetic activity. More recently, Montenegro et al. (2011) showed an increase in HRV following anodal stimulation over the left TC at rest, while Okano et al. (2015) demonstrated a reduction of HR during exercise with increase in HRV during incremental cycling exercise. Both studies associated this behavior with an enhanced para-sympathetic activity. The authors form both studies, suggested that stimulation of the left TC by tDCS can also induce alterations in subcortical brain areas such as the IC given the multiple anatomical connection between these brain areas (Augustine, 1996; Lang et al., 2005).

Contrarily to previous findings, our results did not show any change in cardiovascular response during or after anodal tDCS stimulation. In support of our findings, Vandermeeren et al. (2010) failed to observe any significant variations in HR or blood pressure between anodal, cathodal, or Sham tDCS in healthy subjects at rest, despite significant changes in HRV indexes. It is likely that the inconsistency with previous studies and our current data involving tDCS stimulation can be explained by the different experimental protocol and the variables investigated. For instance, the study of Montenegro et al. (2011) only provides frequency domain parameters of HRV and no functional cardiovascular parameters were presented. Additionally, the study performed by Okano et al. (2015) related the lower HR response following tDCS stimulation during exercise as consequence of an altered activation of the IC. However, given that the test was a graded exercise test to exhaustion, and that participants were able to perform longer in the tDCS condition, it is likely that they were performing at different relative exercise intensities between the two conditions. This could be a possible explanation for the observed differences in HR. Few previous studies investigating the effect of tDCS on the cardiovascular response have been performed (Vandermeeren et al., 2010; Montenegro et al., 2011; Okano et al., 2015), and consequently knowledge regarding the effect of tDCS on the cardiovascular response is limited. A recent meta-analysis by Makovac et al. (2017) investigated the effect of both rTMS and tDCS on autonomic and cardiovascular response by showing that these techniques are effective for reducing HR and enhancing HRV whereas only a marginal effect has been found for blood pressure. These findings, indirectly confirm a potential pathogenic “brain-heart pathway” to cardiovascular disease.

Compounding this, there is a difficulty in interpretation of previous results due to different experimental procedures and sample size. Indeed, three main limitations are present in previous literature: (1) there are no studies comparing tDCS stimulation of both the left and right TC on the cardiovascular parameters. (2) Studies have been performed in the absence of a placebo controlled condition. (3) The cardiovascular parameters investigated and reported are limited and thus the exact effect on the cardiovascular system is uncertain. To address this, we used a PEMI protocol, which provides a unique opportunity to monitor and isolate the two main sympathetic systems regulating the cardiovascular responses (i.e., central command and metaboreflex), thus allowing a more in-depth analysis of any possible changes in cardiovascular response.

Given the growing number of studies involving tDCS prior to exercise (Cogiamanian et al., 2007; Muthalib et al., 2013; Angius et al., 2015, 2017a,b), it is very important to understand its effect on the cardiovascular response as any moderation of this has the potential to effect blood flow to the working muscles, and thus effect exercise capacity. To date, the only parameters used to assess the effect of tDCS on cardiovascular control have been MAP, HR, and measures of HRV. In current experiment, the integration of variables such as SV, HR, and SV/LVET provides a greater opportunity to examine potential tDCS induced change in parasympathetic and sympathetic balance on cardiac regulation. Okano et al. (2015) found a significant reduction in HR during the first phases of a maximal incremental exercise test following anodal tDCS over left TC. Unfortunately, given the nature of the test performed, this protocol is unlikely to be appropriate for the monitoring of tDCS effect due to the changes in exercise intensity. Indeed, a maximal incremental test implies a continuous increase in power output, which requires an increase in sympathetic drive to increase cardiac response to satisfy oxygen demand of the working muscles. These rapid changes in cardiovascular dynamics make interpretation of the effect of tDCS unclear. Rather, a constant load exercise should be performed to reduce these methodological limitations. The handgrip exercise performed during the PEMI in the current study was executed at the same absolute and relative workload. Taken together, the setup used in the current study should be able to better monitor any cardiovascular changes induced by tDCS administration, with less methodological constraints.

The application of non-invasive brain stimulation can potentially become a useful tool for the treatment of various chronic cardiovascular or autonomic disorders. In this regard, Cogiamanian et al. (2010) suggested that rTMS and tDCS can provide a novel therapeutic tool for human arterial hypertension, and Sampaio et al. (2012) highlighted the possibility to treat cardiovascular autonomic complications induced by stress. These conditions can cause near-fatal and fatal arrhythmias which can potentially lead to sudden unexpected death. The same non-invasive brain stimulation techniques could be applied in the specific field of exercise rehabilitation in chronic respiratory disorders. One of the main issues in such diseases are the breathing discomfort during exercise and the difficulty to induce significant fatigue following a given training session (Gruet, 2018). In this regards Nierat et al. (2015) demonstrated that rTMS can reduce breathing pattern in healthy individuals. A similar intervention could be used to alleviate the negative effects of breathing discomfort and possibly increase exercise tolerance. There is potential for these techniques to treat cardiovascular and respiratory disorders, however, the acute and chronic effect needs to be established more firmly.

The results of the current study suggest that an acute bout of tDCS stimulation over the left and right TC has no effect on functional cardiovascular parameters in a normal, healthy population, and that it is likely to have little impact on cardiovascular response if applied during/before exercise. Further research should seek to identify whether the use of tDCS is effective in treating individuals with cardiovascular disorders.

### Limitations

One of the limitations of the present study is that we did not monitor a surrogate of autonomic control of the heart such as HRV, instead only measuring functional parameters of cardiovascular activity. Therefore, we cannot surely affirm that tDCS stimulation did not induce changes in the autonomic control of cardiovascular response, despite observing that it did not appear to change the functional parameters. A second limitation is that the brain areas involved in cardiovascular regulation, such as the IC, has a deep location and might not have been reached by tDCS. However, it should be considered that the tDCS montage used for the present investigation has been previously demonstrate to induce changes in autonomic regulation of the heart (Montenegro et al., 2011; Okano et al., 2015). Furthermore, a computational model of brain current flow during tDCS applied over T3, demonstrated its ability to reach deep brain areas such as IC (Okano et al., 2015). It should be noted that several other potential factors might had influenced the efficacy of the stimulation and consequently our experimental findings such as, genetics, neural structure, or thickness of bone (Ridding and Ziemann, 2010). Stimulation parameters such as duration and intensity might play an important role in affecting the targeted brain area (Ridding and Ziemann, 2010; Batsikadze et al., 2013). Further studies are required to elucidate neurophysiological mechanisms and to optimize tDCS protocols.

## Conclusion

Although the key brain areas related to autonomic cardiovascular control have been well-established, the literature regarding the use of non-invasive brain stimulation techniques to modulate autonomic regulation demonstrate a lack of consistency in findings (Cogiamanian et al., 2010). The results of the current study suggest that anodal tDCS of the left and right TC does not affect functional cardiovascular response at rest, during exercise and PEMI. It should be taken into account that the number of studies investigating the effect of non-invasive stimulation such as tDCS of the cardiovascular response in very limited and therefore further experiments are needed to confirm the results of the current study. However, in light of the present and previous findings, the effect of tDCS on the cardiovascular response remains inconclusive.

## Ethics Statement

This study was carried out in accordance with the recommendations of ‘School of Sport and Exercise Sciences Research Advisory Group, University of Kent,’ with written informed consent from all subjects. All subjects gave written informed consent in accordance with the Declaration of Helsinki. The protocol was approved by the ‘School of Sport and Exercise Sciences Research Advisory Group.’

## Author Contributions

LA, SM, and AM were involved in planning the experiment. LA performed the measurements, processed the experimental data, performed the analysis, drafted the manuscript, and designed the figures. SM, JH, and AM aided in interpreting the results and worked on the manuscript.

## Conflict of Interest Statement

The authors declare that the research was conducted in the absence of any commercial or financial relationships that could be construed as a potential conflict of interest.
